# Burn Selection: How Fire Injury Shaped Human Evolution

**DOI:** 10.1002/bies.70109

**Published:** 2026-02-04

**Authors:** Joshua Cuddihy, Yuemin Li, Isobel Fisher, Zoltan Takats, Dominic Friston, Declan Collins, Marcela Vizcaychipi, Matteo Fumagalli, Istvan Nagy, Armand Leroi

**Affiliations:** ^1^ Nociception group, Division of Anaesthetics, Pain Medicine and Intensive Care, Department of Surgery and Cancer Imperial College London, UK; ^2^ Chelsea and Westminster Hospital NHS Foundation Trust London UK; ^3^ School of Biological and Behavioural Sciences Queen Mary University of London, UK; ^4^ Department of Life Sciences Imperial College London London UK; ^5^ Department of Surgery and Cancer Imperial College London London UK

**Keywords:** fire use, human evolution, inflammation, natural selection, thermal trauma, wound healing

## Abstract

The mastery of fire transformed human evolution through advantages spanning diet, behavior, physiology, and ecology. While these benefits are well established, here we highlight a previously overlooked cost — and selective pressure — unique to humans: high‐temperature burn injury. Unlike other species, humans and their hominin ancestors have faced increased lifetime risk of burns, which we argue has driven genetic adaptation. Drawing on comparative genomic evidence across primates, we suggest that genes associated with burn injury response — relating to wound healing and inflammation — show signs of accelerated evolution in humans. We propose that recurrent exposure to burns acted as a selective force in our lineage, helping to explain both beneficial adaptations and paradoxical maladaptive responses to severe injury. By framing burns as an evolutionary pressure, the *Burn Selection Hypothesis* invites a re‐evaluation of how fire shaped human biology and offers new perspectives for understanding both the evolutionary past and modern burn care.

## Graphical abstract

Intentional fire use exposed humans and their ancestors to high‐temperature burn injury, a risk rare in other species, bringing major survival benefits and technologies but also repeated exposure to extreme heat. The Burn Selection Hypothesis reframes this cost of fire mastery as a unique selective pressure, which shaped our evolution.

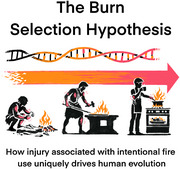

## 1 The Burn Selection Hypothesis

For at least one million years, humans — and our extinct relations — have controlled fire [5, 26, 34]. It is often said that this ability was an important factor in our success [33, 34, 92, 111]. The mastery of fire has influenced human culture in many ways. Amongst other key adaptations, it allowed our species to expand into dark and cold climates, enhanced our diet, facilitated tool development and use, and changed the way we sleep and socialize [33, 51, 109, 111, 58]. Many fundamentally human technologies — ceramics, metallurgy, and alcohol distillation — depend on it. The consequences of controlled fire are intimately involved in modern human life.

The mastery of fire may have also driven adaptive responses in human morphology and physiology. Many of these are thought to be associated with cooking. The reduction in jaw size and dentition observed as early as approximately 1.5 mya (million years ago) [109, 110, 111] might be the result of eating cooked food as the predigestion of foods allowed far greater extraction of nutrients with less time spent chewing and lower risk of pathogen ingestion; the evolution of increased brain size (early *Homo erectus* fossils dated to 1.8 mya indicate brain volumes nearly twice that of modern chimpanzee, modern humans with nearly 3 times the size by 200 kya [thousand years ago]) [28, 34]. We cannot be sure whether the evolution of these features, visible in the fossil record, was driven solely by the use of fire [32, 94], but the enormous survival advantage of controlled fire moving forwards is well recognized. However, whatever the benefits of fire for our species, its use must have entailed costs, too — most obviously the increased risk of burn injuries from proximity to extreme heat.

**Significance Statement**

High‐temperature burn injury — an inevitable consequence of fire use — represents an overlooked evolutionary pressure unique to the human lineage. By reframing burns as an evolutionary pressure, we highlight a perspective with implications not only for modern burn care but also for anthropology and our understanding of human evolution.

Most humans who survive to adulthood will burn themselves at least once, usually mild, occasionally severe. The following statistics give a sense of the risks. Across the globe in 2004, thermal burns severe enough to require medical attention were responsible for nearly 11 million injuries. More than 180 000 of those burns were fatal [108]. The risk of minor burns that do not require medical attention and are, therefore, not recorded must be far greater. (While writing this paper, one of the authors burnt his lower lip — painfully, ludicrously, but not fatally — while biting into a Chicken Kiev filled with molten butter. This is an all‐too‐common injury type, as humans frequently will handle and drink recently boiled liquids, capable of significant injury: to our knowledge, no other species on the planet does this. Just one example of the uniqueness of burn injury risk in humans).

Many human burns are not the result of direct contact with fire, but rather the high‐temperature appliances, like kettles, irons, stove‐tops, ovens, hair rollers/straighteners, and car radiators, that pervade modern life and are a consequence of human fire and combustion technologies [6, 13, 64]. New technologies continue to bring new burn risks and are directly or indirectly related to human fire use and combustion. In the last decade, exploding lithium batteries in phones, cars, and e‐scooters [22, 72] have been globally responsible for many fatalities [115]. Given this fraught relationship with fire — and high temperatures generally — we propose that burns have been an important selective agent in human history. Hence, “The Burn Selection Hypothesis”.

## 2 A Brief History of Controlled Fire

**FIGURE 1:**
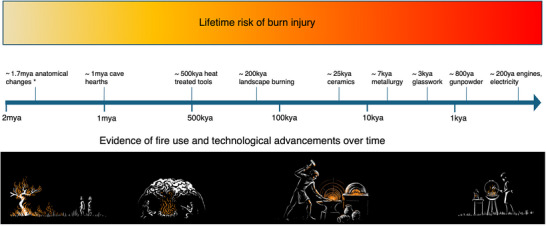
Timeline of hominin adaptations and technologies related to fire use. The figure is presented across a logarithmic timeline from 2 mya (million years ago) to the present. The logarithmic scale reflects the accelerating pace of fire‐related innovations, with more developments occurring in recent millennia than in the preceding millions of years. The yellow to red gradient represents increasing lifetime burn injury risk as humans developed more sophisticated fire technologies. Early evidence appears through anatomical adaptations to new diet (*smaller jaws, increasing brain size, and short gut), suggesting cooking practices (1.7 mya) and controlled fire use in caves (1 mya). Technological sophistication increased with heat‐treated stone tools (174–72 kya [thousand years ago]) and landscape burning (400–200 kya), transformative developments in ceramics (25 kya), metallurgy (7 kya), glassworks (5–3 kya), and gunpowder (800 ya [years ago]). The timeline culminates in industrial‐era technologies with electricity and combustion engines (200 ya), illustrating humanity's increasing mastery and dependence on fire‐based technologies. Images along the bottom of the timeline represent a graphical representation of the changing nature of contact with fire over time.

All preindustrial *Homo sapiens* cultures know, or knew, how to *control* fire, by which we mean make, or at least maintain, it [34, 111]. This implies that the use of fires for cooking, warmth and ceremonies, and many other fundamentally human activities has been a part of human life for a very long time (Figure 1). Although the earliest evidence suggestive of anatomical adaptations to fire use for cooking and changing diet is around 2 million years old, the nature of fire tends to leave relatively few archeological traces. The actual first human ancestors to manipulate and live alongside fire may be even earlier [34].

The earliest undisputed evidence for controlled, rather than natural, fires comes from Wonderwerk Cave, South Africa, and dates to approximately 1 mya [5]. The fire‐makers belonged to the Acheulean culture and were either *H. erectus* or *H. heidelbergensis* [5]. Ash deposits containing burned plant materials and bone fragments demonstrate temperatures of 500–700°C, with microscopic evidence indicating sustained, controlled burning. Importantly, these fires were found inside a cave and therefore were unlikely to have a natural cause [5].

A series of archaeological sites shows the increasing use of fire by hominins over time. Qesem Cave, Israel (archaic *H. sapiens* or *H. neanderthalensis*, 400–300 kya), shows hearths, systematic cooking, and the use of compound adhesives requiring precise temperature control at 250–300°C [7, 49, 97]. Pinnacle Point, South Africa (anatomically modern *H. sapiens*, 164 kya), shows intentional heat treatment of silcrete tools at 300‐400°C [12, 85]. Sibudu Cave, South Africa (70 kya), shows even more complex fire‐related technology, including multi‐phase heating of compound adhesives [77, 107].

The progression of pyrotechnology accelerated with the emergence of ceramics at Xianrendong Cave, China (22 kya), requiring controlled temperatures of 700°C [101]. Early exploitation of fossil fuels began in China (coal, 3 kya) [20] and Mesopotamia (bitumen, 2 kya) [91]. Metallurgy developed with copper smelting at Belovode, Serbia (7 kya) [12], requiring temperatures above 1100°C [82]. Bronze production became widespread by 1.2 kya, followed by iron smelting demanding temperatures of 1538°C [52, 83]. Glass production emerged in Mesopotamia (3.7 kya), demanding temperatures of 1500°C and precise cooling control [18]. In the ninth century before common era (BCE), the Chinese mastered rapid combustion when they invented gunpowder [62].

Fires have been part of the daily lives of all humans for hundreds of thousands of years. It was only in the last century that electricity supplanted the naked flame as a source of domestic heat and light — and then only in developed nations. Today, at least 700 million people, most of whom live in Africa, still have the same, intimate, daily contact with fire that our ancestors had.

## 3 Evolution in the Pyrocene

Natural fires, caused by lightning strikes and the like, are relatively rare [33]. As humans learned to control and use it, fire began to shape terrestrial ecologies. Some ecologists even call the Holocene (170 kya to present) the “Pyrocene” [80], in acknowledgement of how pivotal this advance has been to altering the environment. Unsurprisingly, various notable animal species have adapted to fire [73].

Western fence lizards, *Sceloporus occidentalis*, preferentially perch on stalks of burned shrubs that match their dark scales; *Melanophila acuminata* beetles have evolved infrared sensory pits on their thoraxes by which they sense forest fires; *Antechinus* marsupial mice go torpid and hide during fires [47]. Most of these adaptations are defensive; however, some species take advantage of fires. “Firehawks” — various kite or falcon species — migrate toward bush fires to feed on fleeing animals, and even enhance this feeding opportunity by moving smouldering sticks to start new fires in unburnt areas [10]. Savannah chimpanzees, *Pan troglodytes*, take advantage of spontaneous fires to diversify their diets [10, 34, 78, 79]. These behaviors may be genetic adaptations, cultural, or both. Our hominin ancestors may have first come to understand the uses of fire in similar ways.

We do not know whether these adaptations evolved in response to anthropogenic fires. The most famous example of evolution‐in‐action, however, did. Beginning in the 1750s, England's forests turned black with soot. The *carbonaria* allele of *Biston betularia* increased in frequency; after 1955, as the forests became cleaner, it decreased and is now very rare [17]. This is a story about one moth's response to the human use of fire at an industrial scale.

Animals have, therefore, adapted to fire in various ways. But no animal lineage has been exposed to fire and associated extreme temperature as much as ours. It seems likely, then, that humans have also evolved adaptations that mitigate its malign effects. The aryl hydrocarbon receptor (AHR) gene may be an instance of one, as described by Hubbard et al. The modern human version of this gene contains a nonsynonymous substitution not found in Neanderthals or any other primates — one that specifically reduces the binding of polycyclic aromatic hydrocarbons in smoke of the sort produced by fires [41], such that *H. sapiens* had less harm linked to cooking with fire (smoke inhalation) and could therefore extract greater benefit. If AHR is an example of a gene that has evolved in response to increasing human fire exposure, it is surely only one of many. It is likely that other genes, especially those involved in burns responses, are similarly evolving in humans.

## 4 Burns: The Cost of Fire

**FIGURE 2:**
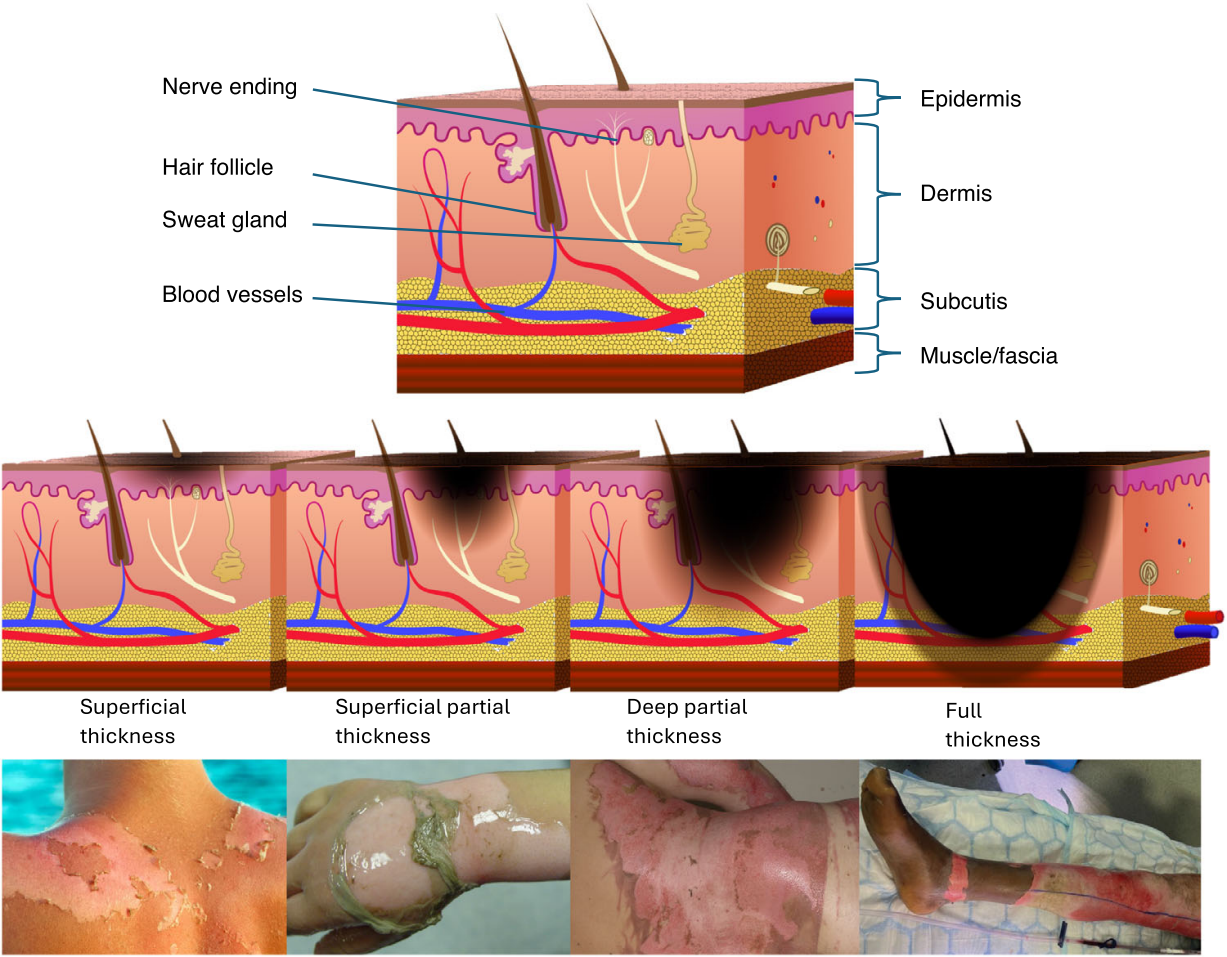
Cross‐sectional anatomy and clinical manifestations of progressive burn depths. Upper image illustrates normal skin anatomy, showing epidermis, dermis (containing hair follicles, sweat glands, nerve endings, and blood vessels), subcutaneous fat, and underlying muscle/fascia. Lower panel, progressing rightward: superficial burns (epidermal only), superficial partial‐thickness (upper dermis), deep partial‐thickness (deeper dermis), and full‐thickness burns (complete dermal destruction). Adjacent clinical photographs demonstrate characteristic appearances: erythema and blistering in superficial/superficial partial burns, moist pink‐white surface in deep partial‐thickness burns, and dry, leathery eschar in full‐thickness injury. Burn depth correlates with progressive destruction of dermal structures [43]. Image (A) adapted from Open Access Government, 2021 [75], Images (B–D) photographs provided with specific patient consent.

Burn injuries result from exposure to high temperatures that cause direct cellular death through necrosis and protein denaturation and coagulation within skin tissues [42, 43, 112]. The severity of thermal damage is determined primarily by the temperature of the heat source and duration of contact. The extent of thermal damage is characterized along two critical dimensions: depth of tissue destruction and total body surface area (TBSA) affected. Depth is classified according to the skin layers involved, with superficial burns affecting only the epidermis, dermal burns involving both epidermis and varying depths of dermis (subdivided into superficial and deep dermal), and full‐thickness burns destroying the entire epidermis, dermis, and underlying subcutaneous structures (Figure 2). The TBSA affected by burns is expressed as a percentage. Injury severity correlates directly with both deeper tissue involvement and larger TBSA, with the combination of these factors strongly influencing both survival probability and healing outcomes. Table 1 gives a classification of burn types [40, 42, 43, 112].

**TABLE 1:** Classification of burns.

Burn depth	Healing time^a^	Scarring	Typical cause
Superficial	3 days	None	Sunburn or flash flame
Superficial partial thickness	4–20 days	Minimal	Flash flame or water‐based scald
Deep partial thickness	>21 days	Significant	Oils scald and flame
Full thickness	Incomplete	Severe	Prolonged flame esp. with accelerant

^a^ Refers to typical healing times but can vary grossly. Causes in the table are typical but not absolute. Severity of injury proportionate to the temperature of initial insult and time of exposure, and each of the main high‐temperature burn mechanisms (scald, contact, and flame) can potentially cause any degree of injury.

The nature and scale of burn injuries fundamentally differ from other forms of skin trauma. Most mechanical injuries, such as lacerations, typically involve discrete, localized areas of tissue disruption with well‐defined margins between damaged and healthy tissue. These injuries trigger precisely regulated inflammatory responses that prioritize rapid hemostasis, wound closure, and defense against pathogen invasion at the injury site. In contrast, burns can affect vast areas of skin, creating extensive zones of tissue damage that preclude rapid approximation of wound edges [59, 105]. Although the initial burn wound is typically sterile for the first 24 hours, due to the thermal destruction of microorganisms, the subsequent loss of skin barrier function across large areas creates a significant risk for infection in the days and weeks that follow. This represents a striking reversal of infectious risk compared to other trauma types [2, 50], where pathogen invasion is an immediate concern requiring rapid inflammatory responses. In the pre‐antibiotic era, infection likely represented the predominant threat to burn survival beyond the acute phase, exerting strong selective pressure for physiological strategies that minimized post‐burn infectious risk.

Burn injuries further differ from other skin trauma in their damage patterns and healing trajectories, creating zones of progressive tissue damage characterized by transitions from central necrosis to peripheral tissue at risk. This pattern, first described by Jackson, presents unique healing challenges requiring specialized cellular responses [25, 40, 43]. Wound progression involves multiple factors including severe inflammation, disrupted blood supply, tissue hypoperfusion, free oxygen radical generation, and programmed cell death [87].

**FIGURE 3:**
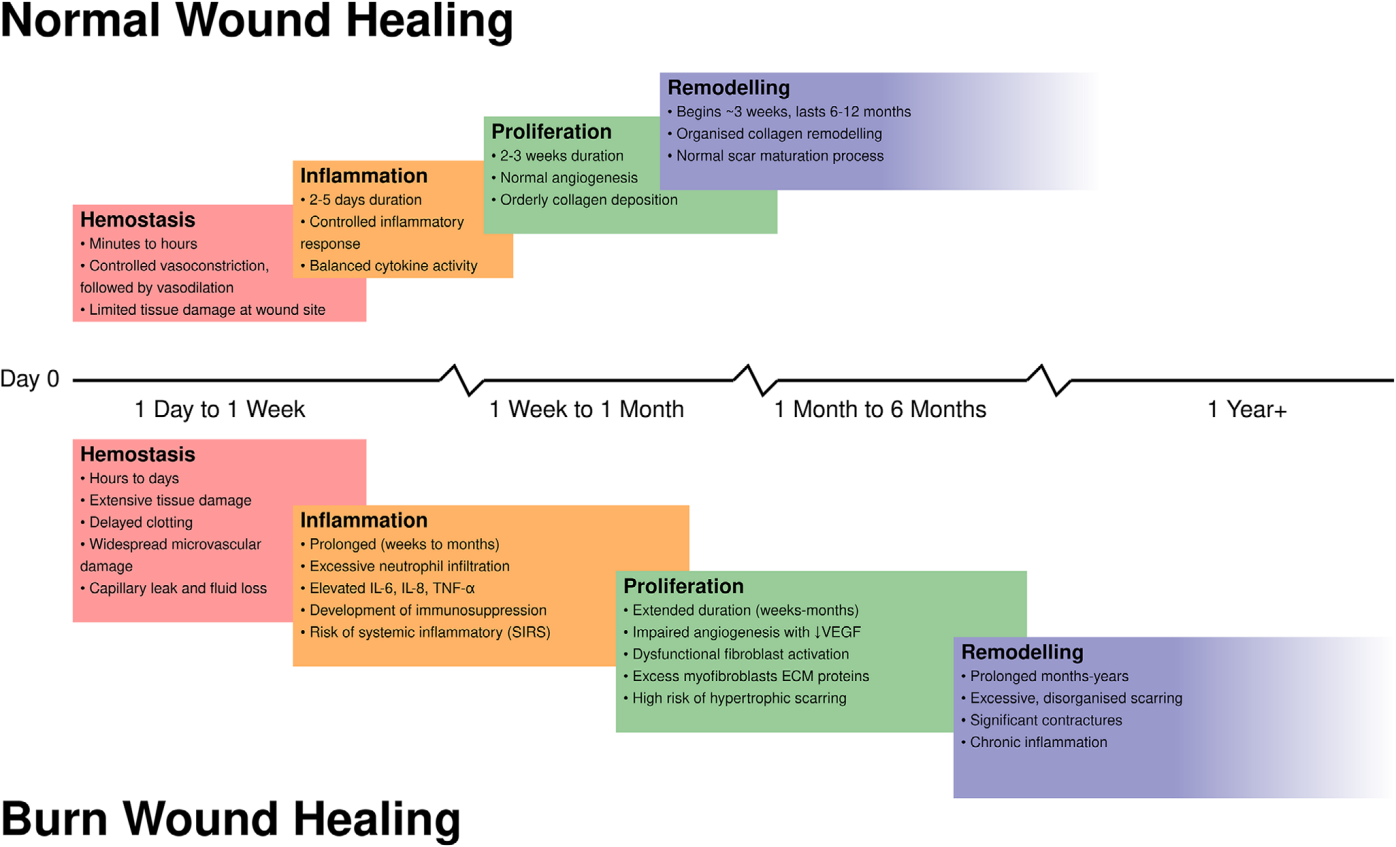
Schematic representation comparing the temporal progression of normal wound healing (top) versus burn wound healing (bottom) across hemostasis, inflammation, proliferation, and remodeling phases. Time points are indicated on the central axis. Overlapping regions between phases indicate temporal overlap, and diagonal hatching in the burn wound remodeling phase indicates the extended period of potential complications and chronic inflammation. Normal wound healing progresses through regulated phases: hemostasis (minutes to hours) with controlled vasoconstriction and vasodilation; inflammation (2–5 days) with balanced cytokine activity; proliferation (2–3 weeks) with normal angiogenesis; and remodeling (6–12 months) with organized collagen remodeling. The timeline depicted represents typical healing for deep dermal burns, with duration varying by burn depth and total body surface area (TBSA). Superficial burns heal faster with fewer complications, while full‐thickness burns and larger TBSA (>20%) experience prolonged healing and increased SIRS risk. Burn wounds show extended phases characterized by extensive tissue damage, excessive inflammation with elevated cytokines, impaired epithelialization, and prolonged remodeling with risk of hypertrophic scarring. Normal wounds have the highest infection risk at injury, whereas burn wounds are initially sterile but become increasingly susceptible during hemostasis and inflammation phases. Wound infections frequently further complicate and prolong burn wound healing.

The burn healing cascade diverges significantly from typical wound resolution (Figure 3). Burns trigger an intensified inflammatory phase with profound mediator and cytokine release, leading to extensive edema, dramatic fluid shifts, and potential systemic inflammatory response syndrome (SIRS) [25, 40, 90, 112, 43, 44].

The proliferative phase is delayed due to extensive damage to skin architecture [25, 40, 90, 112, 43, 44], while destruction of skin appendages in deeper dermal burns impairs tissue regeneration [88], necessitating extensive granulation tissue formation and increasing pathological scarring risk [105, 112].

The remodeling phase can extend beyond a year, sometimes continuing for several years [43], often resulting in hypertrophic scarring and contractures. Joint contractures can cause significant functional loss [43], particularly affecting growing children's development [27, 95]. Healed tissue typically exhibits permanently reduced elasticity and altered mechanical properties.

## 5 The Evolutionary Genomics of Burn Responses

If fire has been an important agent in human history, we should see the evidence of this in our genomes. Following this reasoning, we identified and studied a set of burn injury response genes.

To identify them, we compared the transcriptomes of burnt and unburnt skin. Gene expression responses to burns are complex and very variable, so, to identify the most important genes, we examined two datasets together: one derived from humans and another from rats, filtering for orthologs that showed statistically significant changes in expression, in the same direction, in both datasets. (Orthology refers to genes in different species that evolved from a common ancestral gene by speciation and typically retain equivalent functions). This yielded a set of 94 differentially expressed genes (DEGs) that are likely to be important in mammalian burn responses.

**FIGURE 4:**
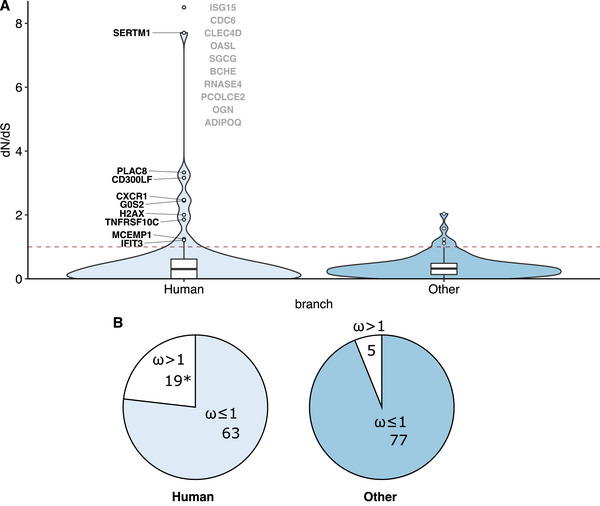
Branch‐specific *d_N_*/*d_S_* (*ω*) ratios. (A) Distributions of *d_N_*/*d_S_* ratios. Two ratios are estimated on the foreground (human) and background branches. Violin plots show the distributions as well as three quartiles and outliers plotted individually as points. The dashed line indicates *d_N_*/*d_S_* = 1. Ten ratios for the human branch are infinities, which can occur in the absence of synonymous substitutions along the branch. Although it is difficult to estimate the exact values of *ω* in such situations, these ratios are plotted on the top but excluded in the violin plots. (B) Proportions of genes with *d_N_*/*d_S_* ratios > 1 and ≤ 1 for both the human and all other branches. *Indicates infinite values included in *d_N_*/*d_S_* ratios > 1 count.

To determine whether these genes have been under selection, we estimated branch specific *d_N_*/*d_S_* ratios (ratio of nonsynonymous [amino acid‐changing] to synonymous [silent] substitutions in protein coding genes — a measure of selective pressure on genes. Values >1 indicate positive selection) in human compared with non‐human primate lineages (chimpanzee, gorilla, orangutan, and macaque). (See Supplementary information). We found that, of the 94 genes, at least nine genes, and potentially 19, showed evidence for positive selection (*d_N_*/*d_S_* > 1) in the lineage leading to humans, of which four showed strong positive selection (*d_N_*/*d_S_* > 2), one of which showed *d_N_*/*d_S_* of >7 (SERTM1) (Figure 4, Table 2). Nielsen et al. [71] and Bustamante et al. [14] estimate approximately 2%–5% of genes show *d_N_*/*d_S_* ratio of >1 across the human and chimpanzee genome. Our results reveal at least 9.5% (9/94) and potentially 20% (19/94) DEGs have a ratio >1 in the human lineage and point to a process beyond chance alone. We propose this is due to specific selection linked to burn injury in the human lineage.

**TABLE 2:** Burn injury response genes with *d_N_*/*d_S_* > 1 selected in the human lineage, ordered in descending *d_N_*/*d_S_*.

Gene name	Protein	Potential burn response	Reference
^a^ *ISG15*	Ubiquitin like protein	Antiviral, immunity, and inflammation regulation	[8]
^a^ *CDC6*	DNA pre‐replication complex	Initiate and regulate DNA replication	[69]
^a^ *CLEC4D*	Calcium dependent PRR	Pathogen detection on myeloid cells	[37]
^a^ *OASL*	2′‐5′‐Oligoadenylate synthase	Infection and inflammation signaling	[55]
^a^ *SGCG*	Gamma‐sarcoglycan	ECM structure and metabolism	[53]
^a^ *BCGE*	Enzyme butyrylcholinesterase	Toxin metabolism	[67]
^a^ *RNASE4*	Ribonuclease 4	RNA metabolism and host defense	[98]
^a^ *PCOLCE2*	PCPE‐2	ECM regulation and immune response	[4]
^a^ *OGN*	Osteoglycin	Collagen formation and tissue remodeling	[74]
^a^ *ADIPOQ*	Adiponectin	METABOLIC and lipid regulation	[48]
*SERTM1*	Intracellular membrane protein	Unknown	[70]
*PLAC8*	Placenta‐specific 8	Immune regulation and cytokine production	[117]
*CD300LF*	Immune cell receptor	Modulates inflammation	[11]
*G0S2*	G0/G1 switch gene 2	Lipid metabolism and apoptosis	[39]
*CXCR1*	Chemokine receptor	Innate immune inflammation	[89]
*H2AX*	H2A.X variant histone	DNA damage response	[9]
*MCEMP1*	Type 2 transmembrane protein	Immune response and monocyte function	[76]
*TNFRSF10C*	Decoy TRAIL receptor	Regulates apoptosis	[68]
*IFIT3*	Interferon‐induced protein	Antiviral, immunity, and apoptosis regulation	[31]

Abbreviations: ECM = extracellular matrix, PRR = pattern recognition receptor, TRAIL = tumor necrosis factor‐related apoptosis‐inducing ligand.

^a^ Indicates those genes with *d_N_*/*d_S_* of infinity due to the absence of synonymous substitutions along the branch.

We have identified a set of genes that are; (i) associated in mammals with burn injury responses; (ii) have been under directional selection in the human lineage only. We take this as preliminary evidence that humans have been selected for an increased ability to respond to burns. The genes that we identified are associated with reducing wound progression and faster healing, although some genes may be further linked to disordered functional recovery, that is, scar tissue.

PLAC8, CD300LF, and CXCR1 notably play key roles in inflammation, immunity, infection response, and tissue healing. PLAC8 encodes a small, cysteine‐rich protein expressed in a range of human tissues. It aids in the differentiation of immune cells, including macrophages and neutrophils, and aids in the response and clearance of intracellular bacteria [117]. CD300LF (Cluster of Differentiation 300‐like Family Member F) is a transmembrane inhibitory receptor primarily expressed on myeloid cells and some dendritic cells. It plays a critical role in dampening immune responses, maintaining immune homeostasis, and preventing excessive inflammation [11, 60]. CXCR1 encodes a G‐protein‐coupled receptor that binds interleukin‐8 family chemokines, driving neutrophil and macrophage recruitment — critical for pathogen clearance and early immune responses [61, 66]. Evolutionarily, selection for these genes may have conferred a survival advantage by optimizing early immune responses, enhancing tissue repair, and mitigating infection risks following burns — an injury likely in early human populations due to fire use and environmental hazards.

All of these genes may be involved in general tissue damage responses. Thus, we cannot exclude other selective agents such as injuries from tool use or fighting. However, burns differ from other kinds of skin trauma in the size and degree of immediate skin loss, and the relative sterility of the initial wound. As the genomic basis of burn responses becomes clearer and more distinct from the responses to other kinds of trauma, we will be able to test the Burn Selection Hypothesis with a larger and better defined set of genes.

## 6 An Evolutionary View of Burns

The Burn Selection Hypothesis belongs to the tradition of Darwinian medicine, which takes an evolutionary view of disease [106]. Twenty years ago, cancer was viewed mostly as a disease; now it is understood to be a selective agent that has shaped the molecular devices that regulate cell behavior [56, 102]. In the same way, here we draw attention to an under‐appreciated selective agent that has shaped the human body. We argue that the intentional use of fire by humans and our hominin ancestors — a behavior virtually absent in other species — created an unprecedented lifetime risk of burn injuries over millennia, with fire‐using hominins and modern humans having immune systems that evolved differently over tens of thousands of generations in response to this selective pressure. This risk likely drove specific genetic adaptations related, but not limited, to, wound closure, inflammation, infection responses, and pain responses, though these likely represent only a subset of the total adaptations involved. Although we present evidence of positive selection in several genes associated with burn injury responses, we acknowledge the inherent complexity of burn pathophysiology. Many of the genetic adaptations we discuss participate in broader inflammatory and healing processes beyond thermal injuries alone. Our aim is not to comprehensively catalog all burn‐related adaptations, but rather to establish the theoretical framework that burn injury constitutes a significant and previously underappreciated selective force in human evolution.

## 7 Possible Adaptations via Burn Selection

### 7.1 Burn Selection Emphasizes Significant Benefit of Fire Mastery

The profound risk of death and severe trauma associated with burn injuries has shaped the evolutionary responses of most organisms, typically manifesting as instinctive avoidance behaviors [46]. However, humans and their hominin ancestors diverged remarkably from this pattern, repeatedly engaging with fire despite its potentially devastating consequences. This exceptional behavioral adaptation suggests that the evolutionary cost‐benefit equation strongly favored fire interaction, implying that the advantages conferred by fire use — including cooking, protection, and thermal regulation — must have been sufficiently substantial to outweigh the considerable risks of injury and mortality. This striking example of risk‐reward trade‐off in human evolution underscores the transformative role that fire mastery played in our species’ development, where the benefits were so profound that they superseded one of nature's most fundamental survival imperatives.

### 7.2 The Uniqueness of Human Skin

Human skin's distinctive anatomical features, including increased dermal thickness and high density of cutaneous appendages, especially sweat glands [3], may represent evolutionary adaptations to thermal injury risk. The evolution of human skin has already been linked to several adaptive advantages — the reduction in body hair facilitating thermoregulation through enhanced sweating, sexual selection, social bonding, bipedalism, and infant care [99]. Beyond these evolutionary adaptations, humans possess a thicker dermis with deeply positioned hair follicles and eccrine sweat glands [3], potentially providing enhanced protection during thermal injury. Although mechanical injuries heal through linear re‐epithelialization, burn injuries require a distributed healing mechanism, where preserved deep dermal appendages serve as epithelial stem cell repositories enabling multiple foci of re‐epithelialization across the wound bed [45, 116]. The survival of these deeper structures, along with their associated commensal microbiota [86], supports efficient wound regeneration even in partial‐thickness burns, suggesting that protection from thermal injury may represent another adaptive advantage alongside thermoregulation, social behavior, and locomotion in shaping the unique characteristics of human skin. Of note, this dermal‐based reservoir for healing is lost in full‐thickness injuries, pointing toward healing strategies after smaller burns being most selected for.

### 7.3 Scarring

Hypertrophic scarring and contractures following burn injury present significant clinical challenges [54, 105]. We propose that this scarring represents an evolutionary trade‐off, where intense inflammation and rapid wound closure evolved to prevent infection at the cost of functional limitation. Scar patterns differ between species and, notably, are most prominent in larger species [16], with humans displaying a particular predisposition to hypertrophic scarring [19, 103]. We suggest this trade‐off is heightened in burn injuries due to selective pressure for accelerated barrier restoration following thermal injury.

### 7.4 Burn Pain Phenomenon

Burn injury induces significant pain, a primary focus for improving modern burn care [65, 105]. Post‐burn heat hypersensitivity, where burned skin has a lower thermo‐nociception threshold, is a key feature [65]. Small burns are associated with lower morbidity and mortality compared to large burns. Post‐burn heat hypersensitivity, whilst extremely unpleasant, is likely to increase vigilance to further heat sources, thus reducing the risk of further burn injury and creation of a larger and more life‐threatening wound. As such, we suggest the mechanisms behind post‐burn heat hypersensitivity are positively selected for and are of survival advantage. Variations in thermosensation across species, including differences in the activation thresholds of transient receptor potential ion channel subfamily V member 1 (TRPV1) channels and post‐injury thermal sensitivity, suggest species‐specific adaptations to environmental conditions [21, 35, 63, 96]. Although baseline heat pain thresholds appear relatively conserved across mammals (typically 42–45°C) [100, 104], the nature and extent of post‐burn hypersensitivity may vary depending on differences in TRPV1 channel expression. This could initiate action potential generation via TRPV1 and the voltage gated channel Nav1.7 at lower temperatures, as well as other tissue‐level changes associated with burn‐induced inflammation [93]. It remains unclear, however, whether burn injuries elicit more pronounced heat hyperalgesia than other types of skin injury, such as incisions, across species, or whether human sensory neurons show greater sensitivity to burn‐specific inflammatory mediators. The unique ecological and behavioral relationship between hominins and fire raises the possibility that fire exposure may have influenced thermal sensory pathways in distinct ways, though direct evidence for human‐specific evolutionary adaptations in thermoreceptor function remains limited.

### 7.5 Selection Likely Favors Healing From Smaller Injuries

In the context of human evolutionary history, the selective pressures exerted by burn injuries likely operated predominantly through small to moderate thermal injuries rather than severe burns, with important implications for understanding our adaptive responses. For burn selection to be effective, burnt individuals must survive to reproduce, which was likely the case throughout human history given that nearly all burns were then, as now, relatively small (*≤*20% TBSA). Critical or extensive burns would have been nearly uniformly fatal in pre‐modern medical environments, regardless of social support structures. Without the specialized medical interventions that emerged only within the past 70–80 years — including fluid resuscitation, infection control, surgical debridement, and skin grafting — individuals suffering major burns would rarely survive long enough to reproduce and parent offspring to maturity. Severe burns (>40% TBSA) carry high mortality rates even today, often due to multiple organ failure, SIRS, sepsis, or respiratory complications [13, 25, 43], with the Baux score (age + TBSA%) remaining a reliable predictor where scores exceeding 100 historically indicated likely death [84]. Even smaller burns posed significant threats to survival, primarily through infection in the pre‐antibiotic era.

Consequently, the genetic adaptations we observe today likely reflect selection for recovery mechanisms optimized to heal from smaller burns, with particular emphasis on preventing infection and rapidly restoring skin architecture. We suggest that the potential adaptations identified are devices for limiting the number, depth, and extent of small burns, and for promoting their rapid healing. The robust inflammatory response that characterizes human burn healing likely represents an adaptation that was beneficial for limited thermal injuries but becomes maladaptive when scaled to extensive burns — an evolutionary paradox where intense activation could fight infection and promote wound closure in smaller injuries, crucial advantages before antibiotics, and modern surgery.

This evolutionary perspective suggests that modern humans are descended from individuals who possessed sufficient genetic resilience to recover from thermal injuries and successfully contribute to subsequent generations. We propose that genes involved in burn response, particularly those governing wound progression and inflammatory cell function, have been under selection in the hominin lineage for at least 1 million years, with selection pressure likely intensifying as advancing fire technologies increased high‐temperature exposure risk. This pattern of selection helps explain both the likely positive adaptations to heal from smaller burns and the paradoxically overwhelming inflammatory response to major burns observed in contemporary clinical settings.

### 7.6 Implications for the Use of Animal Models

Much experimental work on tissue and systemic responses to burn injury uses various models of burn injury in animals, frequently rat, pig, and mouse [1]. Pig skin is the closest in terms of anatomical similarity to human skin, but retains significant differences, and it is acknowledged that finding the ideal animal model of skin for burn research is not achieved [30]. Further, primates, our closest living relatives, show markedly different skin anatomy, with thinner dermis, fewer deep‐seated hair follicles, and sweat glands [3], and they heal with less collagen deposition compared with humans [36]. The Burn Selection Hypothesis suggests the tissue responses in humans, having been exposed to markedly different selective pressure from fire, are different from non‐human animals. This may explain the significant challenge in finding models of human burn injury that correlate with our own, while non‐burn healing models are more firmly established [36]. Other non‐human species will have ancestors unlikely to have been frequently (if ever) exposed to burn injury, and tissue responses will have evolved in the relative absence of this selective pressure. We suggest that an appreciation of the likely genomic differences in tissue responses to burn injury should be considered when translating animal models of burn injury to humans.

## 8 Conclusion

Burn injury represents a unique trauma type in humans, unlike that of any other species, and is inseparably linked to our intentional control and use of fire. We propose the *Burn Selection Hypothesis*: that the increased risk of burns acted as a selective force in the human lineage, shaping aspects of our genomes, physiology, and healing responses. The specific genes and mechanisms we highlight are not exhaustive, but together they illustrate how fire exposure may have left a lasting evolutionary imprint. Framing burns as an evolutionary pressure offers new insight into human evolution and raises provocative questions about the trade‐offs embedded in our immune and wound‐healing responses. It also points toward new opportunities for connecting evolutionary biology with clinical practice, helping us to understand better both the paradoxes of severe burn pathology and the strategies that may improve treatment in the future.

## Author Contributions

**Joshua Cuddihy**: conceptualization, literature review, data analysis, manuscript writing, and figure preparation. **Yuemin Li**: genomic analysis, methodology development, figure preparation, data interpretation, and manuscript writing. **Isobel Fisher**: genomic analysis. **Zoltan Takats**: supervision and manuscript review. **Dominic Friston**: data analysis and transcriptomic methodology. **Declan Collins**: clinical input, supervision, and manuscript review. **Marcela Vizcaychipi**: supervision, clinical insight, and manuscript review. **Matteo Fumagalli**: genomics supervision and analysis, figure preparation, and manuscript review. **Istvan Nagy**: co‐conceptualization, evolutionary neurobiology input, manuscript review and writing, and supervision. **Armand Leroi**: co‐conceptualization, theoretical development, and manuscript writing. All authors contributed to the final manuscript and approved it for submission.

## Funding

This research received no specific grant from any funding agency in the public, commercial, or not‐for‐profit sectors.

## Ethics Statement

Ethics approval was not required for this study as it is a review article with secondary analysis of publicly available data.

## Consent

Written informed consent was obtained from the patients for the use of clinical photographs in this manuscript. The images are non‐identifiable and are published with full permission for academic and scientific dissemination.

## Conflicts of Interest

The authors declare no conflicts of interest.

## Supporting information




**Supporting File**: bies70109‐sup‐0001‐SuppMat.docx.

## Data Availability

The datasets analyzed during this study are publicly available in the Gene Expression Omnibus (GEO) under accession numbers GSE8056 and GSE102811. Comparative genomic data were obtained from Ensembl (https://www.ensembl.org). All other data supporting the findings of this study are available within the article and its Supporting Information. Analysis scripts will be made available via GitHub upon publication.
