# Supplementary material for: Burn Selection: How Fire Injury Shaped Human Evolution
Source: Bioessays. 2026 Feb 4;48(2):e70109. doi: 10.1002/bies.70109 (PMC12873521; doi:10.1002/bies.70109)
Supplement: Supplementary file 1 — Supporting File: bies70109‐sup‐0001‐SuppMat.docx. [file BIES-48-e70109-s001.docx]

# Supplementary Information

## Materials and Methods

**Human vs rat burn transcriptome analysis**

To identify those genes likely involved in skin response to burn injury, the post-burn transcriptome of experimental burn injury to rat were compared to the post-burn transcriptome of human burn patients. Human microarray expression profiles were obtained from the Gene Expression Omnibus (GEO), with the ascension number GSE8056 [[38](#_bookmark41), [24](#_bookmark27)]. This dataset contained 60 samples, including 45 samples from burn patients and 15 control samples from patients who underwent cosmetic procedures that included excess skin removal. Geo2R on the GEO website was used to compare the 0-3 days post burn groups to the 3 control groups. This microarray data was obtained using the Affymetrix Human Genome U133 Plus 2 Array (AffymetrixInc).

Data from rat experimental burn models previously performed by our group were examined for this study. Details of the processes are described in Friston, D. et al. Elevated 18:0 lysophosphatidylcholine contributes to the development of pain in tissue injury. PAIN 164(2):p e103-e115, February 2023. | DOI: 10.1097/j.pain.0000000000002709 [[29](#_bookmark32)]. Experiments on animals used were performed in accordance with the United Kingdom Animals (Scientific Procedures) Act 1986, the revised National Institutes of Health Guide for the Care and Use of Laboratory Animals, the Directive 2010/63/EU of the European Parliament and the Council on the Protection of Animals Used for Scientific Purposes and the guidelines of the Committee for Research and Ethical Issues of IASP published in PAIN, 16 (1983) 109 to 110. Animals (Sprague Dawley rats) were purchased from Charles River UK. Good Laboratory Practice and ARRIVE guidelines were observed, and all animal procedures were approved by veterinary services (Central Biological Services) at Imperial College London, United Kingdom. Following experimental procedures, animals were terminally anaesthetised at 3 hours after burn using pentobarbital (0.3 mg/kg i.p). 4 rats were scalded, producing a deep partial-thickness burn. Skin samples ipsilateral and contralateral to the burn were collected 3 hours post burn. The rat microarray profiles are deposited at GEO: GSE102811. The contralateral skin samples acted as the control[[29](#_bookmark32)].

To compare the human expression changes with the rat expression changes, the orthologs in each dataset needed to be matched. The human-rat orthologous pairs were downloaded from the HGNC Comparison of Orthology Predictions (HGNC, 2021 [[114](#_bookmark117)]). To match the orthologs, the Affymetrix probe IDs in the human dataset had to be converted to Entrez gene IDs. This was performed in R software (version 4.0.5) using the Bioconductor hgu133a2 package [[15](#_bookmark18), [81](#_bookmark84)]. Matching of the orthologs was also performed in R. This produced a list of 21,957 orthologous pairs which were plotted in R. To determine the correlation strength of the expression changes in these pairs, the Pearson’s correlation coefficient was calculated, also in R. A set of 110 orthologous pairs that were significantly differentially expressed (jlog2fold change (FC)j > 1.5) in the same direction was selected for further analysis. After removing duplicates in the list of human genes, this created a dataset of 94 genes that is referred to as the DEG (differentially expressed genes) dataset (Table 3).


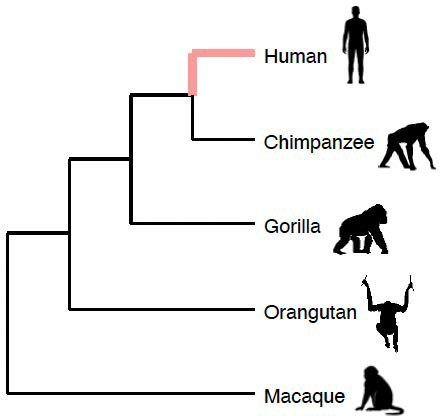


Figure 5: **Phylogenetic tree showing species used in the positive selection analysis using PAML.** The foreground branch is highlighted. The unrooted tree was used for the analysis, whereas here the tree is rooted for clarity.

**Estimating branch-specific** *d_N_ /d_S_* **(***ω***) ratios >1.**

Coding sequences of five primate species for the 94 genes were retrieved from Ensembl using biomaRt [[23](#_bookmark26)], which were restricted to only protein-coding and canonical sequences. We kept only orthologous genes where all five primate species were present (82 out of 94 DEG genes). Sequences were aligned using PRANK with default parameters [[57](#_bookmark60)]. We then estimated *d_N_ /d_S_* ratios (*ω*) using the branch model of codeml from PAML (model = 2; NSsites = 0; fix_omega = 0; clock = 0) [[113](#_bookmark116)]. An unrooted tree is used ((((Human 1, Chimpanzee), Gorilla), Orangutan, Macaque);), specifying the human branch as the foreground branch and all other branches on the tree as the background branches. Two (*ω*) ratios were estimated for each gene: one for the human branch and the other for the remaining primate branches, to detect whether the human branch evolving under positive selection. The direction and magnitude of selection can be measured by the (*ω*) ratio, which indicates positive selection, neutral evolution, and purifying selection, with the respective values of > 1, = 1, <1.

**Data availability**

Human microarray expression profiles were obtained from the Gene Expression Omnibus (GEO), with the ascension number GSE8056. The rat microarray profiles are deposited at GEO: GSE102811. All other data used in this study is from public databases including En- sembl [(http://www.ensembl.org)](http://www.ensembl.org/) and 1000 Genomes Project [(http://www.1000genomes.org).](http://www.1000genomes.org/) Scripts to reproduce statistical analyses will be made publicly available on suitable repositories (e.g., GitHub).

Table 3: List of Differentially Expressed Genes (DEGs) with corresponding branch specific *d_N_ /d_S_* ratio

| Gene | Human *d_N_ /d_S_* | Other *d_N_ /d_S_* |  | Gene | Human *d_N_ /d_S_* | Other *d_N_ /d_S_* |
| --- | --- | --- | --- | --- | --- | --- |
| MMP1 | 0.766463 | 0.228266 |  | PADI4 | 0.178395 | 0.323979 |
| PROK2 | 0.0001 | 1.57919 |  | SGCG | 999.0 | 0.282479 |
| PPBP | 0.0001 | 0.465741 |  | HLA-DQB2 | N/A | N/A |
| IL1B | 0.342571 | 0.396911 |  | SERTM1 | 7.7058 | 0.268103 |
| FPR1 | 0.345088 | 0.678916 |  | FIBIN | N/A | N/A |
| SELL | 0.0001 | 0.326883 |  | MMP27 | 0.489937 | 0.392716 |
| SPP1 | 0.621685 | 0.417776 |  | IGDCC4 | 0.273494 | 0.247259 |
| PLAC8 | 3.33216 | 0.397141 |  | TNN | 0.464147 | 0.2789 |
| TREM1 | 0.419086 | 2.0387 |  | GSTA3 | N/A | N/A |
| MGAM | N/A | N/A |  | FNDC1 | 0.382544 | 0.27613 |
| UPP1 | 0.0001 | 0.0629104 |  | CFD | 0.20755 | 0.299867 |
| MCEMP1 | 1.24967 | 0.993448 |  | AADAC | 0.262089 | 0.483065 |
| FCAR | 0.249487 | 1.1322 |  | CIDEA | 0.309526 | 0.406075 |
| FCGR3B | 0.883461 | 1.23612 |  | BCHE | 999.0 | 0.3551 |
| SLC2A3 | 0.0001 | 0.189186 |  | PLIN4 | N/A | N/A |
| CSF3R | 0.296005 | 0.378283 |  | LPL | 0.104158 | 0.0510317 |
| G0S2 | 2.44723 | 0.420134 |  | SUSD5 | 0.829032 | 0.365091 |
| CR1 | 0.492794 | 0.532878 |  | MB | 0.0001 | 0.128229 |
| FCN1 | 0.610714 | 0.681809 |  | RNASE4 | 999.0 | 0.2296 |
| RSAD2 | 0.448063 | 0.476524 |  | PDGFRL | 0.343786 | 0.244776 |
| CD177 | N/A | N/A |  | AR | 0.634116 | 0.106074 |
| TNFRSF10C | 1.85359 | 0.826056 |  | CFH | 0.413899 | 0.84349 |
| CXCR2 | 0.0001 | 0.654489 |  | PRKAR2B | 0.359356 | 0.132983 |
| MNDA | N/A | N/A |  | CCN5 | 0.0001 | 0.373506 |
| ITGAX | 0.530306 | 0.559719 |  | PCOLCE2 | 999.0 | 0.217837 |
| SELE | 0.187435 | 0.315751 |  | AADACL2 | 0.30195 | 0.223345 |
| FPR2 | N/A | N/A |  | ELOVL4 | 0.0001 | 0.32745 |
| TGM2 | 0.0001 | 0.141536 |  | CKM | 0.0001 | 0.0196522 |
| ULBP2 | N/A | N/A |  | RCAN2 | 0.0001 | 0.0902095 |
| ISG15 | 999.0 | 0.467983 |  | MFAP4 | 0.0001 | 0.0804842 |
| RHCG | 0.616504 | 0.173094 |  | MYL1 | 0.0001 | 0.13971 |
| NEFL | 0.0001 | 0.018389 |  | PLIN1 | 0.911174 | 0.257396 |
| CDC6 | 999.0 | 0.371614 |  | BTC | 0.0001 | 0.743604 |
| CD300LF | 3.16253 | 0.635492 |  | NR3C2 | 0.729851 | 0.114159 |
| SIRPB1 | N/A | N/A |  | NPY1R | 0.0001 | 0.0157019 |
| PILRA | 0.924734 | 0.861008 |  | IGSF10 | 0.1951 | 0.514024 |
| GZMB | 0.67426 | 1.10991 |  | ANGPTL1 | 0.404923 | 0.0828913 |
| FGR | 0.0001 | 0.177757 |  | MYH2 | N/A | N/A |
| H2AX | 2.00451 | 0.0001 |  | DPT | 0.0001 | 0.107968 |
| IRF7 | 0.474024 | 0.342883 |  | ACTA1 | 0.0001 | 0.0160183 |
| CLEC4D | 999.0 | 0.888693 |  | OGN | 999.0 | 0.106607 |
| GINS2 | 0.0001 | 0.0726587 |  | P2RY14 | 0.0893806 | 0.230228 |
| CXCR1 | 2.47166 | 0.485895 |  | CHRDL1 | 0.0001 | 0.0967662 |
| IFIT3 | 1.20867 | 0.428394 |  | PAMR1 | 0.174839 | 0.118498 |
| CCL4 | N/A | N/A |  | MAMDC2 | 0.216148 | 0.0980373 |
| JAK3 | 0.0001 | 0.0804703 |  | CLCA4 | 0.330548 | 0.671304 |
| OASL | 999.0 | 0.560686 |  | ADIPOQ | 999.0 | 0.296107 |

*Legend:* List of Differentially Expressed Genes (DEGs) with corresponding branch specific *d_N_ /d_S_* ratio. Note, N/A indicates there was not 1:1 orthologous genes in all 5 primate species for this analysed gene. 82/94 genes therefore presented with *d_N_ /d_S_* values alongside. Value of 999 representative of "infinite" for purposes of this table.
